# Alterations in Brain-Derived Neurotrophic Factor in the Mouse Hippocampus Following Acute but Not Repeated Benzodiazepine Treatment

**DOI:** 10.1371/journal.pone.0084806

**Published:** 2013-12-19

**Authors:** Stephanie C. Licata, Nina M. Shinday, Megan N. Huizenga, Shayna B. Darnell, Gavin R. Sangrey, Uwe Rudolph, James K. Rowlett, Ghazaleh Sadri-Vakili

**Affiliations:** 1 McLean Hospital, Belmont, Massachusetts, United States of America; 2 New England Primate Research Center, Southborough, Massachusetts, United States of America; 3 Massachusetts General Hospital, Charlestown, Massachusetts, United States of America; 4 Harvard Medical School, Boston, Massachusetts, United States of America; University of Colorado, United States of America

## Abstract

Benzodiazepines (BZs) are safe drugs for treating anxiety, sleep, and seizure disorders, but their use also results in unwanted effects including memory impairment, abuse, and dependence. The present study aimed to reveal the molecular mechanisms that may contribute to the effects of BZs in the hippocampus (HIP), an area involved in drug-related plasticity, by investigating the regulation of immediate early genes following BZ administration. Previous studies have demonstrated that both brain derived neurotrophic factor (BDNF) and c-Fos contribute to memory- and abuse-related processes that occur within the HIP, and their expression is altered in response to BZ exposure. In the current study, mice received acute or repeated administration of BZs and HIP tissue was analyzed for alterations in BDNF and c-Fos expression. Although no significant changes in BDNF or c-Fos were observed in response to twice-daily intraperitoneal (i.p.) injections of diazepam (10 mg/kg + 5 mg/kg) or zolpidem (ZP; 2.5 mg/kg + 2.5 mg/kg), acute i.p. administration of both triazolam (0.03 mg/kg) and ZP (1.0 mg/kg) decreased BDNF protein levels within the HIP relative to vehicle, without any effect on c-Fos. ZP specifically reduced exon IV-containing BDNF transcripts with a concomitant increase in the association of methyl-CpG binding protein 2 (MeCP2) with BDNF promoter IV, suggesting that MeCP2 activity at this promoter may represent a ZP-specific mechanism for reducing BDNF expression. ZP also increased the association of phosphorylated cAMP response element binding protein (pCREB) with BDNF promoter I. Future work should examine the interaction between ZP and DNA as the cause for altered gene expression in the HIP, given that BZs can enter the nucleus and intercalate into DNA directly.

## Introduction

Benzodiazepines (BZs) and related drugs such as zolpidem increase GABA-mediated inhibition via positive allosteric modulation of GABA_A_ receptors throughout the central nervous system [1]. This drug class is commonly prescribed for treating anxiety, sleep, and seizure disorders, and while clinically valuable, their use can result in undesirable effects including memory impairment as well as abuse and dependence [2,3]. Given their widespread application, understanding more fully how BZs produce their effects is an important public health issue that will provide a framework for designing novel compounds to overcome their limitations as therapeutics. 

 A recent study using functional imaging to visualize global drug action within the brain suggested that alterations in coordinated brain activity within networks of brain regions may underlie the changes in observable behavior induced by BZ-like drugs [4]. Meanwhile, the cell surface interactions between BZs and specific subtypes of the GABA_A_ receptor has been shown to be critically important for determining the behavioral response to these drugs [5]. Together, network and receptor mechanisms contribute to our understanding of how BZs affect behavior, but there is a gap in our knowledge regarding the molecular substrates mediating the effects of this drug class. 

Previous studies have provided the foundation for examining the influence of BZs on intracellular processes and signaling cascades by showing that proteins involved in regulating synaptic function and plasticity are sensitive to BZ challenge [6-8]. Accordingly, changes in immediate early gene expression [9-14] have been observed following BZ treatment. Brain-derived neurotrophic factor (BDNF) and c-Fos are of particular interest in this regard given that both are reduced by BZ exposure [6,9,15-17], although contradictory results have been reported [18]. Further, both are implicated in learning- [19,20] and drug abuse-related [21-23] neuronal plasticity. Together, their importance in brain function and the modulation of their expression by BZs, suggests that examining BDNF and c-Fos may provide insight that will be informative for clarifying the molecular mechanisms of BZ action.

The present study investigated the regulation of BDNF and c-Fos following administration of the BZs triazolam (TZ) and diazepam (DZ), as well as zolpidem (ZP), which is structurally distinct but BZ-like in its mechanism of action. It was hypothesized that understanding how administration of BZ-like drugs affects immediate early gene expression would reveal potential points of intervention for influencing the regulation of key proteins as strategies for avoiding or ameliorating the limiting effects BZs. Acute and repeated drug challenges were employed to replicate and extend previous findings [6,9-17]. Results indicated that while there was a significant reduction in BDNF protein in the hippocampus (HIP), an area involved in drug-related plasticity [24], there was no change in c-Fos levels. Consequently, the study focused on BZ-induced regulation of the BDNF gene. 

## Methods

### Ethics Statement

These studies were approved by the Institutional Animal Care and Use Committees of the Harvard University Medical School (Protocol 04184) and McLean Hospital (#11-10/2-6), and they were conducted according to the Guide for the Care and Use of Laboratory Animals (NIH publication no. 85–23, revised 1996).

### Animals

A total of 134 (62 acute and 72 repeated) male C57BL/6J (four to six weeks of age) were group housed in a temperature- and humidity- controlled facility with a 12 hour light/dark cycle (lights on at 7AM). All animals were provided with water and food *ad libitum*. Mice were handled and habituated to the housing room for at least one week prior to drug treatment. Mice in the acute treatment group were randomly assigned to receive a single injection of TZ (0.03 mg/kg), ZP (1.0 mg/kg), or vehicle (VEH; 80% propylene glycol/20% sterile water) [25], and they were sacrificed within 30 min of treatment [26]. Those mice in the repeated treatment group were randomly assigned to receive twice-daily injections (10 am and 4 pm) of DZ (10 mg/kg + 5 mg/kg), ZP (2.5 mg/kg + 2.5 mg/kg), or VEH (10% cyclodextrin in sterile water) over the course of seven consecutive days [27,28], and they were sacrificed 60 min after the last injection. All injections were via the intraperitoneal route and in a volume of 0.01 ml/g. At the conclusion of the injection paradigm, animals were euthanized with CO_2_ followed by decapitation. After sacrifice all brains were removed quickly and the hippocampi were dissected. Tissue was flash frozen using 2-methylbutane and maintained at -80° C. 

### Western Blotting

Whole cell extracts from dissected brain tissue were used for blots as described previously [29,30]. Briefly, 20 μg of each sample was boiled in the presence of sample buffer for 5 min before separation on 10-20% SDS-polyacrylamide gel. Proteins were transferred to nitrocellulose membranes. The immunoblots were blocked with 5% nonfat dry milk dissolved in Tris-buffered saline containing 0.2% Tween 20 (TBST) for 60 min. The membranes were incubated overnight at 4° C with specific antibodies including anti-BDNF 1:700 (Aviva Systems Biology), anti-TrkB 1:600 (Santa Cruz), anti-cFos 1:400 (Abcam), anti- phosphorylated cAMP response element binding protein (pCREB, Ser 133) 1:500 (Millipore), anti-CREB 1:700 (Abcam), anti-di-acetyl lysine 9, lysine14 histone H3 (H3K9K14Ac2; AcH3) 1:1000 (Millipore), anti-Methyl-CpG binding protein 2 (MeCP2) 1:500 (Abcam), and anti-GAPDH 1:1000 (Abcam). Primary antibody incubation was followed by six washes (10 min, rocking at room temperature) in TBST before incubation with the secondary antibody (HRP-conjugated goat anti-rabbit IgG; Jackson ImmunoResearch Laboratories), followed by visualization using the enhanced chemiluminescence detection system (NEN). 

### Enzyme-Linked Immunosorbant Assay (ELISA)

Brain tissue was lysed, homogenized, and diluted to 20 μg/μl in order to quantify the concentration of BDNF using the *Chemikine*™ BDNF Sandwich ELISA kit (Chemicon International Inc.). The tissue samples and serial dilutions of BDNF standards were loaded in triplicate onto a microplate coated with rabbit anti-mouse BDNF polyclonal antibodies and incubated overnight at 4° C. After four washes, biotinylated mouse anti-mouse BDNF monoclonal antibody (1:1000) was added for 2.5 h at room temperature. The plates were washed four times, strepavidin-enzyme conjugate was added, and plates incubated for 1 h. After further washing, tetramethylbenzidine chromagenic substrate was added and the reaction was stopped after 15 min. The absorbance at 450 nm was measured with a plate reader, and BDNF concentration in the tissue was assessed by comparing values to the prepared standard curve.

### RNA Extraction and Reverse Transcription

RNA was extracted using the RNeasy kit (Qiagen) according to manufacturer’s instructions and as described previously [29,30]. Reverse transcription reactions were performed using the Superscript First Strand Synthesis System for RT-PCR reactions (Invitrogen) using specific primers to quantify the amount of gene expression as compared to a standard curve. Primers included GAPDH and BDNF as previously published sequences [30-32]. Quantitative real time-PCR was performed in an iCycler (Bio-Rad) using SYBR-green PCR Master Mix (Applied Biosystems) through 50 PCR cycles (95° C for 30 s, 57° C for 60 s, 72° C for 90 s). The threshold cycle for each sample was chosen from the linear range and converted to a starting quantity by interpolation from a standard curve run on the same plate for each set of primers. Levels of mRNA were normalized to GAPDH. 

### Chromatin Immunoprecipitation (ChIP)

The ChIP technique was used to analyze DNA/protein complexes in the dissected brain tissue. Protocols were adapted and modified from previously published studies [33,34] and described extensively in recent work [30,32]. Briefly, the tissue underwent a formaldehyde cross-linking step to link the proteins of interest to DNA. Samples then were homogenized and subjected to immunoprecipitation with 5 µg of antibodies specific for Methyl-CpG binding protein 2 (MeCP2; Abcam), di-acetyl lysine 9, lysine14 histone H3 (H3K9K14Ac2; AcH3; Millipore), or pCREB (Abcam). The antibody-transcription factor-DNA complexes were washed to reverse the crosslinks, and the DNA was detected by qPCR using specific primers for the multiple BDNF promoters. Threshold amplification cycle numbers (T_c_) using iCycler software were used to calculate IP DNA quantities as percentage of corresponding inputs. The exon-specific BDNF primers were designed based on previously published sequences [35-37] and used for real-time PCR analysis. 

### Data Analyses

For western blot analyses integrated density values (% IDV) were calculated by dividing the density values for the protein of interest by the density values for GAPDH for each sample. Transcript levels also were analyzed as a percentage of GAPDH. Data then were analyzed with ANOVAs to examine treatment effects. *Post-hoc* pairwise comparisons were performed with Bonferroni to assess treatment effects at a significance level of *p*< 0.05. 

## Results

A number of measurements were obtained following acute or repeated treatment with two classical BZs, TZ and DZ, as well as the BZ-like hypnotic ZP. All three drugs bind to a site on the native pentameric GABA_A_ receptor that is situated between the γ2 subunit and an α subunit, but while the classical BZs are non-selective and will bind α1, α2, α3, or α5 subunit-containing receptors [38,39], ZP exhibits relatively selective affinity for GABA_A_ receptors containing an α1 subunit [40,41].

**Figure 1:**
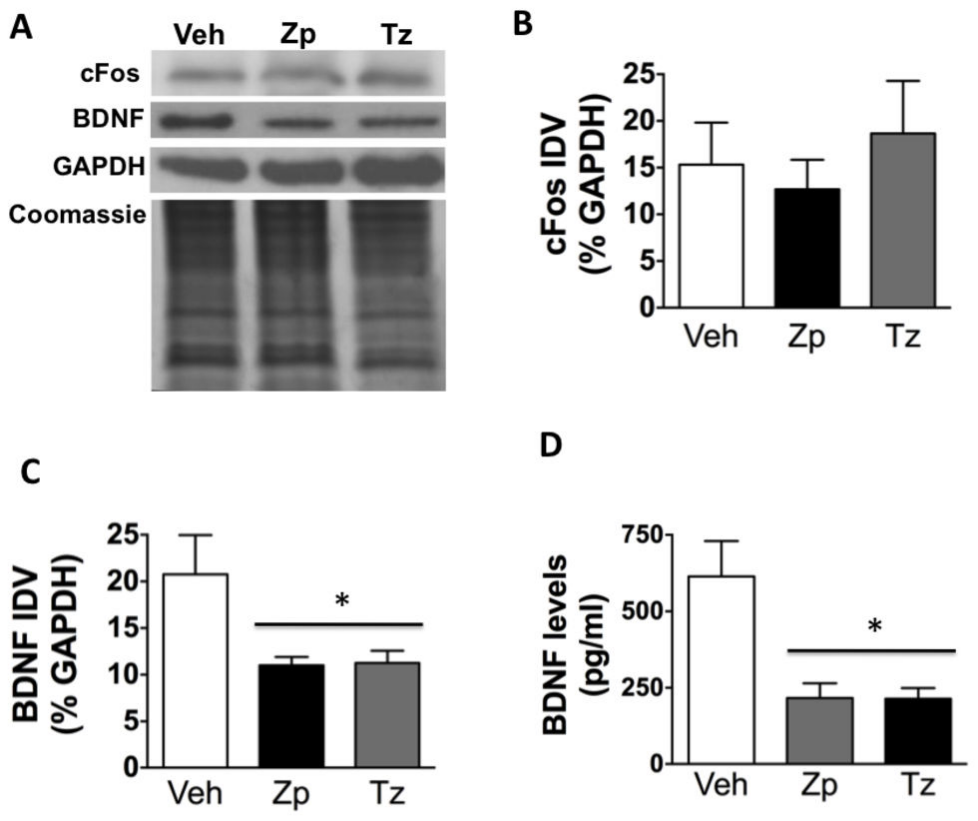
BDNF levels are decreased following acute BZ treatment. (A) Representative immunoblots for c-Fos, BDNF, and GAPDH, as well as Coomassie gel representing the loading control for VEH, ZP, or TZ-treated HIP; (B) There is no change in c-Fos protein levels following BZ treatment as measured by western blots (n= 3). (C) There is a significant decrease in BDNF levels in the HIP following ZP and TZ treatment as measured by western blots (n= 4); (D) There is a significant decrease in BDNF levels following BZ treatment as measured by ELISA (n= 3-6). * *p*< 0.05; IDV: integrated density values.

Levels of c-Fos and BDNF protein were measured by western blots and analyzed with one-way ANOVAs. While acute BZ treatment had no significant effect on c-Fos relative to VEH (Figure 1A & B), there was a significant decrease in BDNF following acute treatment with both ZP and TZ in the HIP [F_(2,9)_= 4.57, *p*= 0.043] (Figure 1A & C). This observation was confirmed with ELISA, which also demonstrated a significant reduction in BDNF protein levels in the HIP following acute ZP and TZ [F_(2,9)_= 9.568, *p*= 0.006] (Figure 1D). 

**Figure 2:**
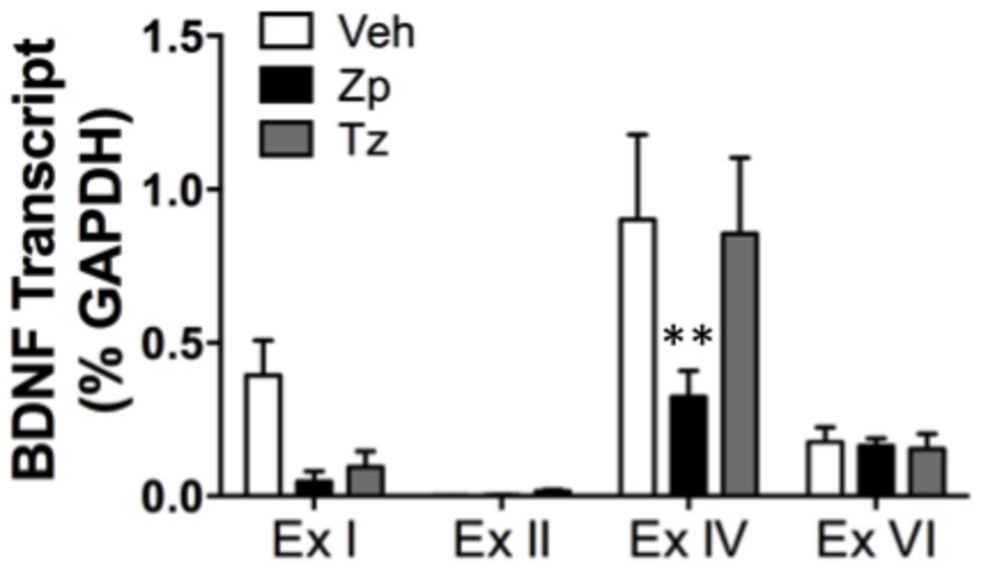
BDNF exon IV-containing transcript is decreased following acute ZP treatment. There is a selective and significant decrease in BDNF exon IV-containing transcript following ZP treatment compared to VEH in the HIP (n= 4-7). ** *p* < 0.01.

The BDNF gene is comprised of eight untranslated 5′ exons that are spliced onto the coding 3′ exon containing the coding domain used to generate multiple mRNAs [31,42,43]. The function of these transcripts is not known, however, their expression pattern differs between brain nuclei and they are differentially targeted and translated within cells. A two-way ANOVA examining the expression of BDNF exon I, II, IV, and VI-containing transcripts in the HIP following BZ treatment indicated that not only do the amounts of the four transcripts vary within this brain region as illustrated by the white bars representing VEH in Figure 2 [F_(3, 55)_= 16.05, *p*< 0.0001], but there was a significant main effect of treatment [F_(2, 55)_= 3.26, *p*= 0.046]. Specifically, there was a significant decrease in BDNF exon IV-containing transcript levels following acute treatment with ZP (*p*< 0.01; Figure 2). 

Because acute BZ treatment reduced BDNF levels in the HIP, the molecular mechanisms by which BZs regulate BDNF expression were investigated. Given that gene expression is influenced by sequence-specific binding of transcription factors to promoter regions, it was hypothesized that BZ-induced decreases in BDNF levels in the HIP resulted from alterations in the assembly of transcription factors at BDNF promoters. BDNF gene expression is regulated by the activity of a number of transcription factors, including CREB and MeCP2 [35,37,44]. Therefore, changes in pCREB (phosphor-Ser133; the activated form of CREB) and MeCP2 in response to acute BZ treatment were measured next. 

**Figure 3:**
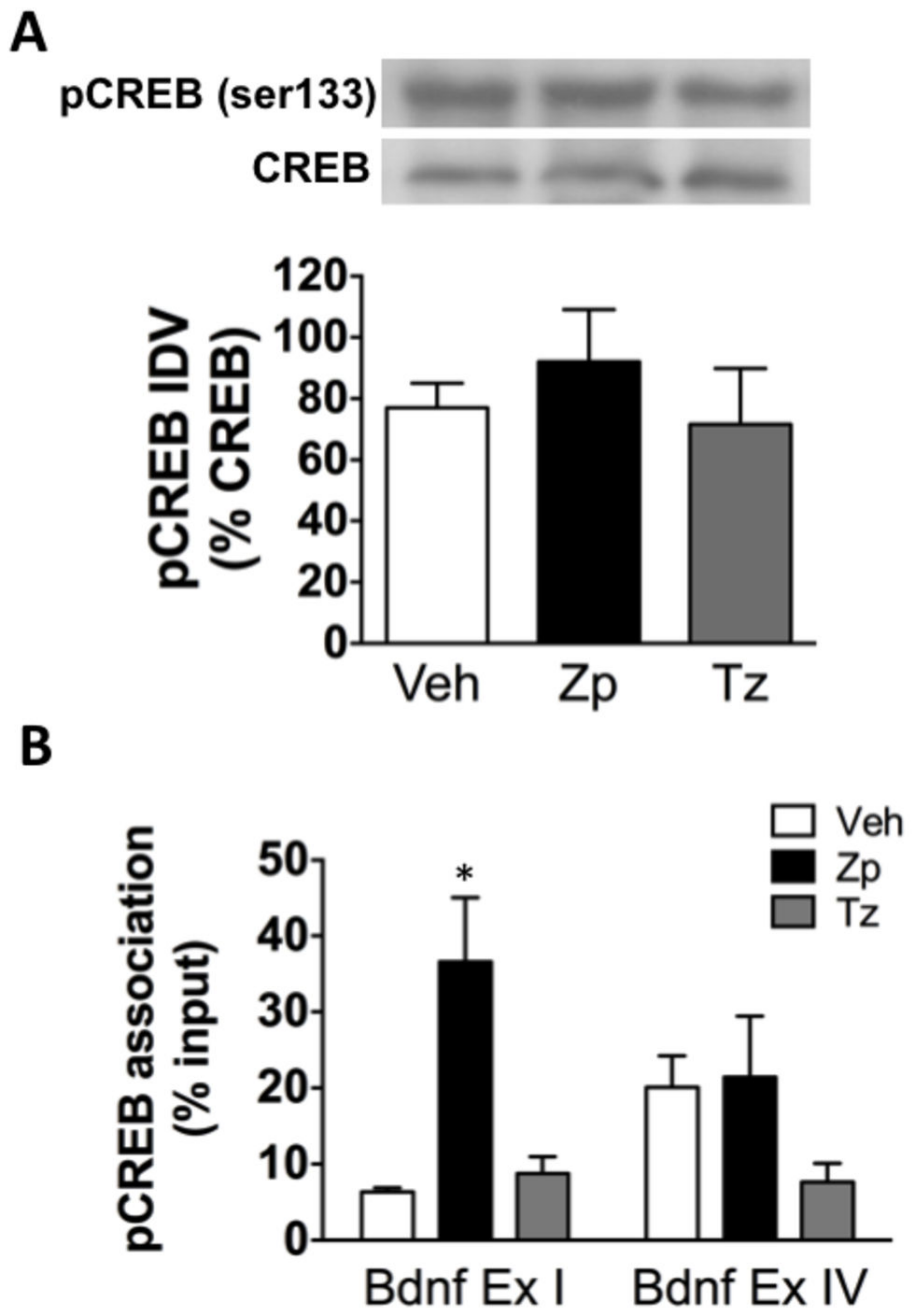
pCREB levels in the HIP following acute BZ treatment. (A) There is no change in total pCREB protein levels in the HIP following BZ treatment as measured by western blots (n= 4); (B) There is a significant increase in pCREB association with BDNF promoter I in the HIP following ZP treatment as measured by ChIP (n= 5-7). * *p*< 0.05; IDV: integrated density values.

CREB is known to regulate the expression of promoter I- and IV-containing BDNF transcripts [35,44], therefore it was predicted that BZs would decrease pCREB. Although one-way ANOVA indicated there were no statistically significant changes in total CREB [F_(2,9)_= 1.99, *p*= 0.183] or overall levels of pCREB (normalized to total CREB) following treatment [F_(2,9)_= 0.134, *p*= 0.877] (Figure 3A), global pCREB levels do not represent action at individual gene promoters. Therefore, ChIP was used to assess alterations in pCREB binding to BDNF promoters. Two-way ANOVA examining drug effects on the association with promoters I or IV indicated a significant increase in pCREB binding to BDNF exon I promoter following ZP treatment [F_(2,27)_= 5.65, *p*= 0.009] such that the association following zolpidem specifically was greater than that for VEH and TZ (*p*< 0.05; Figure 3B). 

**Figure 4:**
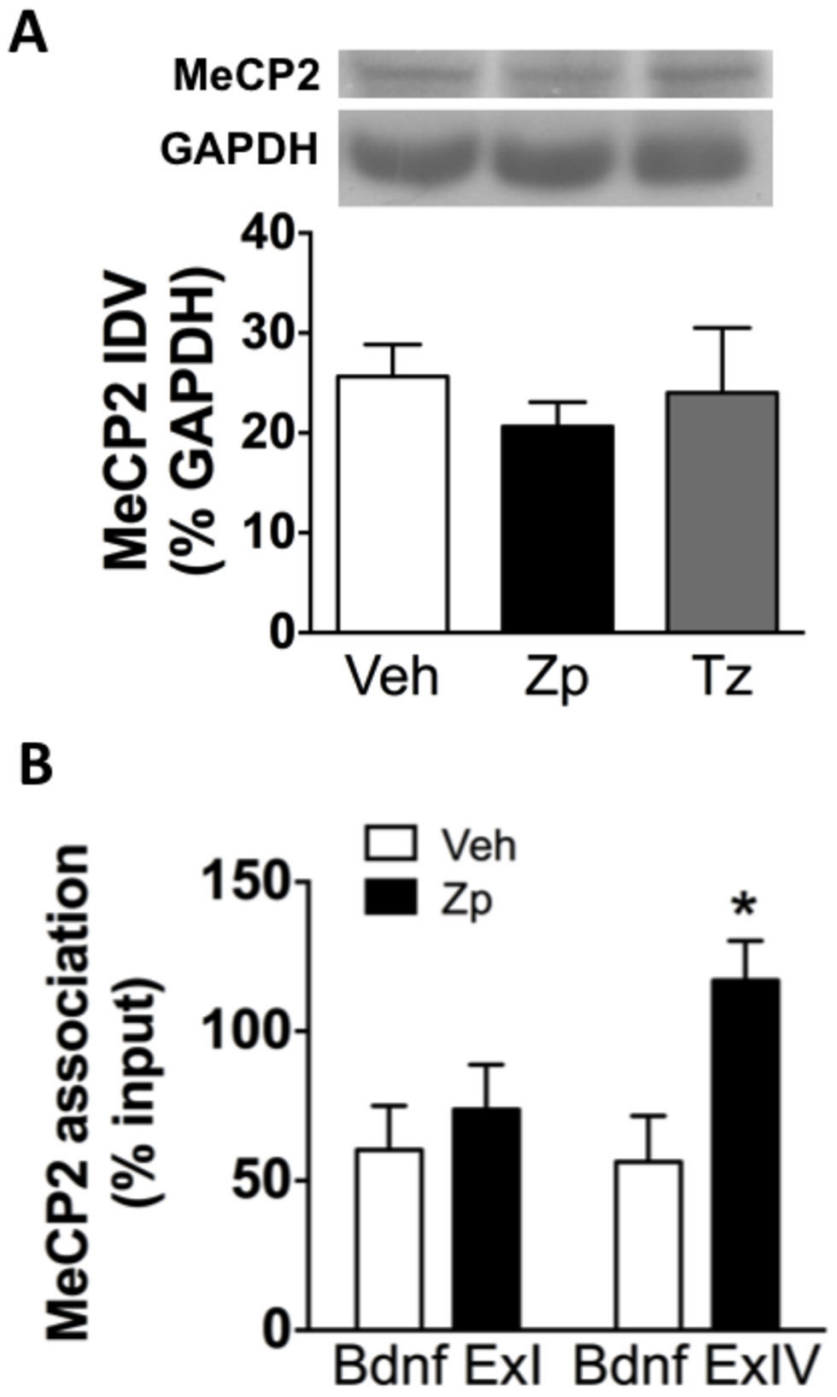
Increased MeCP2 association with BDNF promoter IV in the HIP following acute BZ treatment. (A) There is no change in total MeCP2 protein levels in the HIP following BZ treatment as measured by western blots (n= 3); (B) There is a significant increase in MeCP2 association with BDNF promoter IV in the HIP following ZP treatment as measured by ChIP (n= 3-4). * *p*< 0.05; IDV: integrated density values.

MeCP2 repression of BDNF promoter IV has been well described in previous studies [30,35,37]. A requisite step in BDNF promoter IV-containing gene expression is de-repression caused by the dissociation of MeCP2 from promoter IV [35]. Based on the observed reduction in BDNF promoter IV transcript in response to ZP, it was predicted that ZP would increase MeCP2 association at this specific promoter. One-way ANOVA indicated no change in overall levels of MeCP2 in the HIP [F_(2,6)_= 0.33, *p*= 0.729] (Figure 4A). However, two-way ANOVA revealed a significant increase in the association of MeCP2 with BDNF exon IV promoter in the HIP following acute treatment with ZP as measured by ChIP [F_(1,9)_= 6.09, *p*= 0.036] (Figure 4B). 

**Figure 5:**
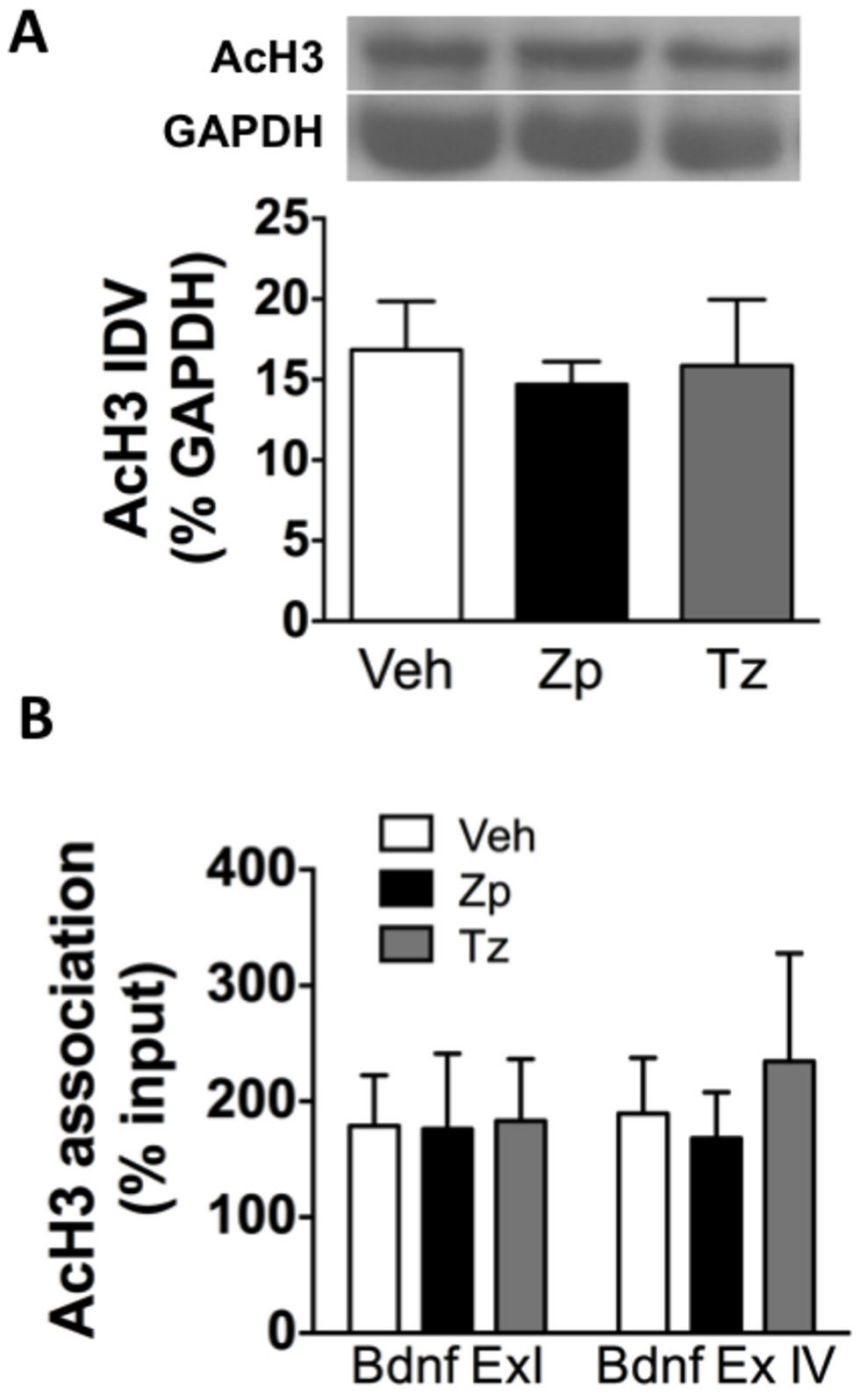
No change in histone acetylation in the HIP following acute BZ treatment. (A) There is no change in total AcH3 protein levels in the HIP following BZ treatment as measured by western blots (n= 3); (B) There is no change in AcH3 association with BDNF promoters in the HIP following ZP treatment as measured by ChIP (n= 4). IDV: integrated density values.

Chromatin remodeling through modification of histone proteins is another requisite mechanism for gene expression. In general, histone acetylation is a modification that relaxes chromatin and corresponds to increased transcription; thus, we predicted that a decrease in acetylation may underlie the observed decrease in BDNF levels within the HIP following acute treatment. As shown in Figure 5A, one-way ANOVA indicated no change in overall AcH3 protein levels in the HIP as measured by western blots [F_(2,6)_= 0.12, *p*= 0.887]. Because global alterations in histone proteins do not necessarily translate into changes in histone association with specific gene promoters, ChIP was used next to assess the extent to which BZ treatment altered AcH3 association with BDNF promoters as a putative mechanism underlying the decrease in BDNF expression in the HIP. The ChIP assay revealed no significant changes in the association of AcH3 with BDNF promoters as two-way ANOVA indicated no significant main effects of treatment (*p*= 0.766) or transcripts (*p*= 0.621; Figure 5B). 

One-way ANOVAs demonstrated that neither acute ZP nor acute TZ had an effect on TrkB receptor [F_(2,6)_= 2.00, *p*= 0.22] protein levels as measured by western blots (data not shown). Moreover, chronic injections of ZP or DZ had no effect on BDNF protein levels compared to VEH [F_(2,21)_= 0.15, *p*= 0.87]. Examination of the activity-related proteins c-Fos [F_(2,12)_= 0.09, *p*= 0.92] and pCREB [F_(2,12)_= 0.89, *p*= 0.44] also indicated no effect of treatment in the repeated condition (data not shown). For this reason MeCP2, AcH3, and TrkB protein, as well as exon-specific mRNA levels, were not examined in the tissue. 

## Discussion

In the present study, acute administration of both TZ and ZP resulted in decreases in BDNF protein levels within the HIP. ZP specifically reduced exon IV-containing BDNF transcripts with a concomitant increase in the association of MeCP2 with BDNF promoter IV, while also increasing the association of pCREB with BDNF promoter I. No significant effects were observed in response to chronic BZ treatment.

 BDNF was hypothesized as a candidate for contributing to the effects of BZs because of its importance in psychoactive drug action in general [45-47] and processes related to abuse and dependence [23] and learning and memory [20] specifically. The data presented here agree with previous findings implicating BDNF in the acute effects of BZs [6,15-17], although repeated drug administration failed to influence BDNF expression. Given that TZ and ZP are known to have deleterious effects on memory when administered acutely [48], a potential explanation for a reduction in BDNF following acute drug administration is that these changes may provide the foundation for drug-induced memory deficits. This interpretation, while speculative without concurrent behavioral outcomes, is consistent with a number of studies showing the blockade of hippocampal long-term potentiation [49-52] or the acquisition of fear conditioning [53 and references therein] by BZs. While ZP once was believed to engender fewer memory impairing effects relative to its non-selective counterparts [54,55], it has since been shown to possess LTP-blocking activity as well [56]. These outcomes are consistent with immunohistochemical data showing that in the HIP there is an abundance of the ZP-specific GABA_A_ receptor subtype (i.e., those containing an α1 subunit) in addition to those with lower ZP binding affinity [57,58], all of which provide binding sites for the classical BZs. 

Another possible interpretation for drug-induced changes in BDNF involves stress-related mechanisms within the brain. Reductions in BDNF [59-61] and mRNA for BDNF exons I and IV [62] have been shown to result from acute exposure to a stressor, suggesting that the inhibitory drug treatment may induce a stress response. While the previously demonstrated reductions in exon-specific mRNA were accompanied by reduced acetylation levels [62] which we did not observe here, the idea of initiating a stress response is consistent with studies showing that BZs and ZP especially, have the ability to activate the hypothalamic-pituitary-adrenocortical axis and elevate plasma corticosterone levels in rodents [63,64]. Moreover, this idea is consistent with work showing that an increase in HIP BDNF associated with a neurogenic antidepressant response [65] was blocked by BZ administration [66,67]. 

Although both acute drug treatments reduced BDNF, they did not exhibit clear effects on pCREB with the exception of the dramatic ZP-induced increase in pCREB association with BDNF promoter I. While this interaction is in agreement with a previous study demonstrating a BZ-induced increase in pCREB levels in mouse brain extract [8], the present result suggests that a drug-activity relationship may exist, perhaps mediated via GABA_A_ receptors containing an α1 subunit specifically. Alternatively, observed effects on pCREB and its association with BDNF promoters may be non-specific and irrelevant to the ability of BZs to modulate BDNF. BZs have been shown to bind to central receptors located within the nucleus [68,69], suggesting that they may be able to bypass the immediate early genes and affect gene expression through a direct interaction with DNA. This idea is supported by a study demonstrating the ability of a BZ to intercalate into DNA, resulting in a more stable and compact DNA conformation [70]. Therefore, it is possible that this type of BZ-induced structural change or interaction may prevent access to the gene and hinder the transcriptional machinery, thus providing a mechanism to explain the reduction in BDNF without the involvement of CREB or histone acetylation. Recently it has been shown that BZs are potent inhibitors of bromodomain and extra-terminal (BET) family of transcriptional co-regulators [71,72]. Bromodomain-containing proteins are epigenetic readers associated with open chromatin architecture and transcriptional activation [73]. Inhibition of BET proteins, therefore, is another potential mechanism whereby BZs can alter the expression of genes such as BDNF. 

Though there were no significant changes in pCREB or acetylated histone association with BDNF promoters in response to acute BZ treatment, there was a significant ZP-induced increase in MeCP2 binding to BDNF exon IV promoter. Given the significant decrease in BDNF exon IV-containing transcript levels following ZP treatment, increases in MeCP2 at this promoter may represent an underlying mechanism whereby ZP specifically reduces BDNF expression. Indeed, previous studies have demonstrated that removal of MeCP2 from BDNF promoter IV is involved in the activity-dependent increase in the expression of BDNF exon IV-containing transcripts [35,37]. Further, NMDA receptor stimulation of cultured HIP neurons increased phosphorylation of MeCP2, its dissociation from the promoter, and increased BDNF exon IV transcript levels [74]. Considering that GABA maintains the balance between excitation and inhibition as evidenced by BZ blocking of NMDA-related activity [75-77], ZP’s ability to enhance GABAergic neurotransmission may oppose glutamatergic activity, thus keeping MeCP2 bound to the BDNF promoter. In addition, increased MeCP2 binding may represent an indirect measure of increases in DNA methylation of CpG islands which are the binding sites for MeCP2. Because MeCP2 binds methylated cytosines, the implication of our finding is that association of MeCP2 to BDNF promoter IV results from a ZP-induced increase in DNA methylation. This idea is speculative, but it provides a rationale for further study. 

In the present study, c-Fos expression was unaltered by either acute or repeated BZ treatment. However, unlike the consistent reduction in BDNF reported in previous work [6,15-17], the expression of c-Fos was not altered reliably by BZs. For instance, while DZ-induced reductions in c-Fos mRNA were observed in the cortex as well as the HIP [6,9], several others reported DZ-induced increases [12,13] or no change in rodent brain tissue [12,78] or cell lines [10]. Considering that a prominent mechanism for influencing transcription of the c-fos gene occurs via a pCREB pathway [79] and pCREB was unchanged here, our results are consistent with one another as well as with the body of literature indicating no early effect of BZs on c-Fos [10,12,78]. 

Examination of intracellular drug-induced alterations was expected to provide further insight regarding the effects of BZs, especially because previous research had implicated several proteins involved in the regulation of synaptic function and plasticity as being important for mediating those effects [6-8,15-17]. However, other work examining GABAergic drugs including muscimol [17,80], bicuculline [81], and ethanol [82], suggests that interpreting the effects on BDNF and exon-specific mRNA may not be attributable to BZs in particular, but modulation of GABA_A_ receptors in general. While our results would provide the foundation for arguing that the modulation of GABA_A_ receptors containing an α1 subunit specifically is important, the overall significance of these inhibition-induced alterations and why they are observed only following acute treatment still is unknown. Future work examining the interaction between ZP and DNA as the cause for altered BDNF expression in the HIP is warranted.

## References

[1] Tallman JF, Thomas JW, Gallager DW (1978) GABAergic modulation of benzodiazepine binding site sensitivity. Nature 274: 383-385. doi:10.1038/274383a0. PubMed: 27722. 27722

[2] Licata SC, Rowlett JK (2008) Abuse and dependence liability of benzodiazepine-type drugs: GABA_A_ receptor modulation and beyond. Pharmacology Biochemistry, and Behavior 90: 74-89. doi:10.1016/j.pbb.2008.01.001. PMC2453238 18295321

[3] Stewart SA (2005) The effects of benzodiazepines on cognition. J Clin Psychiatry 66 [suppl 2]: 9-13. PubMed: 15762814. 15762814

[4] Licata SC, Nickerson LD, Lowen SB, Trksak GH, MacLean RR et al. (2013) The hypnotic zolpidem increases the synchrony of BOLD signal fluctuations in widespread brain networks during a resting paradigm. NeuroImage 70: 211-222. doi:10.1016/j.neuroimage.2012.12.055. PubMed: 23296183. 23296183 PMC3580018

[5] Rudolph U, Möhler H (2006) GABA-based therapeutic approaches: GABAA receptor subtype functions. Curr Opin Pharmacol 6: 18-23. doi:10.1016/j.coph.2005.10.003. PubMed: 16376150. 16376150

[6] Huopaniemi L, Keist R, Randolph A, Certa U, Rudolph U (2004) Diazepam-induced adaptive plasticity revealed by α1 GABA_A_ receptor-specific expression profiling. J Neurochem 88: 1059-1067. doi:10.1046/j.1471-4159.2003.02216.x. PubMed: 15009662. 15009662

[7] Kim DH, Kim S, Jeon SJ, Son KH, Lee S et al. (2009) Tanshinone I enhances learning and memory, and ameliorates memory impairment in mice via the extracellular signal-regulated kinase signaling pathway. Br J Pharmacol 158: 1131-1142. doi:10.1111/j.1476-5381.2009.00378.x. PubMed: 19775283. 19775283 PMC2785534

[8] Saraf MK, Prabhakar S, Pandhi P, Anand A (2008) Bacopa monniera ameliorates amnesic effects of diazepam qualifying behavioral-molecular partitioning. Neuroscience 155: 476-484. doi:10.1016/j.neuroscience.2008.05.043. PubMed: 18585439. 18585439

[9] Bozas E, Tritos N, Phillipidis H, Stylianopoulou F (1997) At least three neurotransmitter systems mediate a stress-induced increase in *c-fos* mRNA in different rat brain areas. Cell Mol Neurobiol 17: 157-169. doi:10.1023/A:1026309727518. PubMed: 9140695. 9140695 PMC11560180

[10] Fukuda K, Shoda T, Mima H, Uga H (2002) Midazolam induces expression of c-Fos and EGR-1 by a non-gabaergic mechanism. Anesth Analg 95: 373-378. PubMed: 12145054. 12145054 10.1097/00000539-200208000-00024

[11] Malkani S, Rosen JB (2000) Differential expression of EGR-1 mRNA in the amygdala following diazepam in contextual fear conditioning. Brain Res 860: 53-63. doi:10.1016/S0006-8993(00)01976-4. PubMed: 10727623. 10727623

[12] Niles LP, Smith LJ, Tenn CC (1997) Modulation of c-fos expression in the rat striatum by diazepam. Neurosci Lett 236: 5-8. doi:10.1016/S0304-3940(97)00755-6. PubMed: 9404938. 9404938

[13] Salminen O, Lahtinen S, Ahtee L (1996) Expression of Fos protein in various rat brain areas following acute nicotine and diazepam. Pharmacol Biochem Behav 54: 241-248. doi:10.1016/0091-3057(95)02132-9. PubMed: 8728564. 8728564

[14] Thompson BL, Rosen JB (2006) Immediate-early gene expression in the central nucleus of the amygdala is not specific for anxiolytic or anxiogenic drugs. Neuropharmacology 50: 57- 68. doi:10.1016/j.neuropharm.2005.07.024. PubMed: 16185722. 16185722

[15] Huang TL, Hung YY (2009) Lorazepam reduces the serum brain-derived neurotrophic factor level in schizophrenia patients with catatonia. Progress in Neuropsychopharmacol and Biological Psychiatry 33: 159-159. 10.1016/j.pnpbp.2008.10.016 19013208

[16] Kellogg CK, Yao J, Pleger GL (2000) Sex-specific effects of in utero manipulation of GABA(A) receptors on pre- and postnatal expression of BDNF in rats. Brain Research. Developmental Brain Research 121: 157-167. doi:10.1016/S0165-3806(00)00039-0. PubMed: 10876028. 10876028

[17] Zafra F, Castrén E, Thoenen H, Lindholm D (1991) Interplay between glutamate and gamma-aminobutyric acid transmitter systems in the physiological regulation of brain-derived neurotrophic factor and nerve growth factor synthesis in hippocampal neurons. Proc Natl Acad Sci U S A 88: 10037-10041. doi:10.1073/pnas.88.22.10037. PubMed: 1658793. 1658793 PMC52862

[18] Balu DT, Hoshaw BA, Malberg JE, Rosenzweig-Lipson S, Schechter LE et al. (2008) Differential regulation of central BDNF protein levels by antidepressant and non-antidepressant drug treatments. Brain Res 1211: 37-43. doi:10.1016/j.brainres.2008.03.023. PubMed: 18433734. 18433734 PMC2394727

[19] Guzowski JF (2002) Insights into immediate-early gene function in hippocampal memory consolidation using antisense oligonucleotide and fluorescent imaging approaches. Hippocampus 12: 86-104. doi:10.1002/hipo.10010.abs. PubMed: 11918292. 11918292

[20] Lu B, Chow A (1999) Neurotrophins and hippocampal synaptic transmission and plasticity. J Neurosci Res 58: 76-87. doi:10.1002/(SICI)1097-4547(19991001)58:1. PubMed: 10491573. 10491573

[21] Chang SL, Squinto SP, Harlan RE (1988) Morphine activation of c-fos expression in rat brain. Biochem Biophys Res Commun 157: 698-704. doi:10.1016/S0006-291X(88)80306-1. PubMed: 3144275. 3144275

[22] Marie-Claire C, Laurendeau I, Canestrelli C, Courtin C, Vidaud M et al. (2003) Fos but not Cart (cocaine and amphetamine regulated transcript) is overexpressed by several drugs of abuse: a comparative study using real-time quantitative polymerase chain reaction in rat brain. Neurosci Lett 345: 77-80. doi:10.1016/S0304-3940(03)00307-0. PubMed: 12821175. 12821175

[23] Russo SJ, Mazei-Robison MS, Ables JL, Nestler EJ (2009) Neurotrophic factors and structural plasticity in addiction. Neuropharmacology 56 Suppl 1: 73-82. doi:10.1016/j.neuropharm.2008.06.059. PubMed: 18647613. PMC2635335 18647613

[24] Eisch AJ, Harburg GC (2006) Opiates, psychostimulants, and adult hippocampal neurogenesis: Insights for addiction and stem cell biology. Hippocampus 16: 271-286. doi:10.1002/hipo.20161. PubMed: 16411230. 16411230

[25] Sanger DJ, Joly D, Zivkovic B (1986) Effects of zolpidem, a new imidazopyridine hypnotic, on the acquisition of conditioned fear in mice. Psychopharmacology (Berl) 90: 207-210. PubMed: 2878460. 2878460 10.1007/BF00181243

[26] Benavides J, Peny B, Dubois A, Perrault G, Morel E et al. (1988) In vivo interaction of zolpidem with central benzodiazepine (BZD) binding sites (as labeled by [3H]Ro 15-1788) in the mouse brain. Preferential affinity of zolpidem for the omega 1 (BZD1) subtype. J Pharmacol Exp Ther 245: 1033-1041. PubMed: 2838599. 2838599

[27] Elliott EE, White JM (2000) Precipitated and spontaneous withdrawal following administration of lorazepam but not zolpidem. Pharmacology Biochemistry, and Behavior 66: 361-369. doi:10.1016/S0091-3057(00)00176-3. 10880691

[28] van Rijnsoever C, Täuber M, Choulli MK, Keist R, Rudolph U et al. (2004) Requirement of alpha5-GABAA receptors for the development of tolerance to the sedative action of diazepam in mice. J Neurosci 24: 6785-6790. doi:10.1523/JNEUROSCI.1067-04.2004. PubMed: 15282283. 15282283 PMC6729721

[29] Sadri-Vakili G, Bouzou B, Benn CL, Kim MO, Chawla P et al. (2007) Histones associated with downregulated genes are hypo-acetylated in Huntington’s disease models. Hum Mol Genet 16: 1293-1306. doi:10.1093/hmg/ddm078. PubMed: 17409194. 17409194

[30] Sadri-Vakili G, Kumaresan V, Schmidt HD, Famous KR, Chawla P et al. (2010) Cocaine-induced chromatin remodeling increases brain-derived neurotrophic factor transcription in the rat medial prefrontal cortex, which alters the reinforcing efficacy of cocaine. J Neurosci 30: 11735-11744. doi:10.1523/JNEUROSCI.2328-10.2010. PubMed: 20810894. 20810894 PMC2943400

[31] Aid T, Kazantseva A, Piirsoo M, Palm K, Timmusk T (2007) Mouse and rat BDNF gene structure and expression revisited. J Neurosci Res 85: 525-535. doi:10.1002/jnr.21139. PubMed: 17149751. 17149751 PMC1878509

[32] Schmidt HD, Sangrey GR, Darnell SB, Schassburger RL, Cha JH et al. (2012) Increased brain-derived neurotrophic factor (BDNF) expression in the ventral tegmental area during cocaine abstinence is associated with increased histone acetylation at BDNF exon I-containing promoters. J Neurochem 120: 202-209. doi:10.1111/j.1471-4159.2011.07571.x. PubMed: 22043863. 22043863 PMC3243782

[33] Braveman MW, Chen-Plotkin AS, Yohrling GJ, Cha JH (2004) Chromatin immunoprecipitation technique for study of transcriptional dysregulation in intact mouse brain. Methods Mol Biol 277: 261-276. PubMed: 15201461. 15201461 10.1385/1-59259-804-8:261

[34] Chen-Plotkin AS, Sadri-Vakili G, Yohrling GJ, Braveman MW, Benn CL et al. (2006) Decreased association of the transcription factor Sp1 with genes downregulated in Huntington’s disease. Neurobiol Dis 22: 233-241. doi:10.1016/j.nbd.2005.11.001. PubMed: 16442295. 16442295

[35] Chen WG, Chang Q, Lin Y, Meissner A, West AE et al. (2003) Derepression of BDNF transcription involves calcium-dependent phosphorylation of MeCP2. Science 302: 885-889. doi:10.1126/science.1086446. PubMed: 14593183. 14593183

[36] Jiang X, Tian F, Du Y, Copeland NG, Jenkins NA et al. (2008) BHLHB2 controls Bdnf promoter 4 activity and neuronal excitability. J Neurosci 28: 1118-1130. doi:10.1523/JNEUROSCI.2262-07.2008. PubMed: 18234890. 18234890 PMC6671398

[37] Martinowich K, Hattori D, Wu H, Fouse S, He F et al. (2003) DNA methylation-related chromatin remodeling in activity-dependent BDNF gene regulation. Science 302: 890-893. doi:10.1126/science.1090842. PubMed: 14593184. 14593184

[38] Pritchett DB, Seeburg PH (1991) Gamma-aminobutyric acid type A receptor point mutation increases the affinity of compounds for the benzodiazepine site. Proc Natl Acad Sci U S A 88: 1421-1425. doi:10.1073/pnas.88.4.1421. PubMed: 1847522. 1847522 PMC51030

[39] Wieland HA, Lüddens H, Seeburg PH (1992) A single histidine in GABAA receptors is essential for benzodiazepine agonist binding. J Biol Chem 267: 1426-1429. PubMed: 1346133. 1346133

[40] Hadingham KL, Wingrove P, Le Bourdelles B, Palmer KJ, Ragan CI, et al. (1993) Cloning of cDNA sequences encoding human alpha 2 and alpha 3 gamma-aminobutyric acidA receptor subunits and characterization of the benzodiazepine pharmacology of recombinant alpha 1-, alpha 2-, alpha 3-, and alpha 5-containing human gamma-aminobutyric acidA receptors. Molecular Pharmacology 43: 970-975. 8391122

[41] Sanna E, Busonero F, Talani G, Carta M, Massa F et al. (2002) Comparison of the effects of zaleplon, zolpidem, and triazolam at various GABA_A_ receptor subtypes. Eur J Pharmacol 451: 103-110. doi:10.1016/S0014-2999(02)02191-X. PubMed: 12231378. 12231378

[42] Liu QR, Lu L, Zhu XG, Gong JP, Shaham Y et al. (2006) Rodent BDNF genes, novel promoters, novel splice variants, and regulation by cocaine. Brain Res 1067: 1-12. doi:10.1016/j.brainres.2005.10.004. PubMed: 16376315. 16376315

[43] Timmusk T, Palm K, Metsis M, Reintam T, Paalme V, Saarma M, Persson H (1993) Multiple promoters direct tissue-specific expression of the rat BDNF gene. Neuron 10: 475-489. doi:10.1016/0896-6273(93)90335-O. PubMed: 8461137. 8461137

[44] West AE, Chen WG, Dalva MB, Dolmetsch RE, Kornhauser JM et al. (2001) Calcium regulation of neuronal gene expression. Proceedings of the National Academy of Sciences U_S_A 98: 11024-11031. doi:10.1073/pnas.191352298. PMC58677 11572963

[45] Alme MN, Wibrand K, Dagestad G, Bramham CR (2007) Chronic fluoxetine treatment induces brain region-specific upregulation of genes associated with BDNF-induced long-term potentiation. Neural Plast: 26496: 26496 PubMed: 18301726. 10.1155/2007/26496 PMC2248427 18301726

[46] McGinty JF, Whitfield TW Jr, Berglind WJ (2010) Brain-derived neurotrophic factor and cocaine addiction. Brain Res 1314: 183-193. doi:10.1016/j.brainres.2009.08.078. PubMed: 19732758. 19732758 PMC2819624

[47] Xu H, Chen Z, He J, Haimanot S, Li X et al. (2006) Synergistic effects of quetiapine and venlaxafine in preventing the chronic restraint stress-induced decrease in cell proliferation and BDNF expression in rat hippocampus. Hippocampus 16: 551-559. doi:10.1002/hipo.20184. PubMed: 16652337. 16652337

[48] Mintzer MZ, Griffiths RR (1999) Triazolam and zolpidem: effects on human memory and attentional processes. Psychopharmacology (Berl) 144: 8-19. doi:10.1007/s002130050971. PubMed: 10379619. 10379619

[49] del Cerro S, Jung M, Lynch G (1992) Benzodiazepines block long-term potentiation in slices of hippocampus and piriform cortex. Neuroscience 49: 1-6. doi:10.1016/0306-4522(92)90071-9. PubMed: 1407540. 1407540

[50] Maubach KA, Martin K, Choudhury HI, Seabrook GR (2004) Triazolam suppresses the inductin of hippocampal long-term potentiation. Neuroreport 15: 1145-1149. doi:10.1097/00001756-200405190-00013. PubMed: 15129163. 15129163

[51] Mori K, Togashi H, Kojima T, Matsumoto M, Ohashi S et al. (2001) Different effects of anxiolytic agents, diazepam and 5-HT(1A) agonist tandospirone, on hippocampal long-term potentiation in vivo. Pharmacol Biochem Behav 69: 367-372. doi:10.1016/S0091-3057(01)00546-9. PubMed: 11509193. 11509193

[52] Xu JY, Sastry BR (2005) Benzodiazepine involvement in LTP of the GABA-ergic IPSC in rat hippocampal CA1 neurons. Brain Res 1062: 134-143. doi:10.1016/j.brainres.2005.09.008. PubMed: 16266690. 16266690

[53] Oliveira DR, Sanada PF, Saragossa Filho AC, Innocenti LR, Oler G et al. (2009) Neuromodulatory property of standardized extract Ginkgo biloba L. (EGb 761) on memory: behavioral and molecular evidence. Brain Res 1269: 68-89. doi:10.1016/j.brainres.2008.11.105. PubMed: 19146837. 19146837

[54] Balkin TJ, O’Donnell VM, Wesensten N, McCann U, Belenky G (1992) Comparison of the daytime sleep and performance effects of zolpidem versus triazolam. Psychopharmacology (Berl) 107: 83-88. doi:10.1007/BF02244970. PubMed: 1589566. 1589566

[55] Evans SM, Funderburk FR, Griffiths RR (1990) Zolpidem and triazolam in humans: behavioral and subjective effects and abuse liability. J Pharmacol Exp Ther 255: 1246-1255. PubMed: 2262904. 2262904

[56] Higashima M, Kinoshita H, Koshino Y (1998) Differences in the effects of zolpidem and diazepam on recurrent inhibition and long-term potentiation in rat hippocampal slices. Neurosci Lett 245: 77-80. doi:10.1016/S0304-3940(98)00178-5. PubMed: 9605489. 9605489

[57] Pirker S, Schwarzer C, Wieselthaler A, Sieghart W, Sperk G (2000) GABA_A_ receptors: immunocytochemical distribution of 13 subunits in the adult rat brain. Neuroscience 101: 815-850. doi:10.1016/S0306-4522(00)00442-5. PubMed: 11113332. 11113332

[58] Sperk G, Schwarzer C, Tsunashima K, Fuchs K, Sieghart W (1997) GABA(A) receptor subunits in the rat hippocampus I: immunocytochemical distribution of 13 subunits. Neuroscience 80: 987-1000. doi:10.1016/S0306-4522(97)00146-2. PubMed: 9284055. 9284055

[59] Fuchikami M, Morinobu S, Kurata A, Yamamoto S, Yamawaki S (2009) Single immobilization stress differentially alters the expression profile of transcripts of the brain-derived neurotrophic factor (BDNF) gene and histone acetylation at its promoters in the rat hippocampus. Int J Neuropsychopharmacol 12: 73-82. doi:10.1017/S1461145708008997. PubMed: 18544182. 18544182

[60] Schaaf MJ, de Jong J, de Kloet ER, Vreugdenhil E (1998) Downregulation of BDNF mRNA and protein in the rat hippocampus by corticosterone. Brain Res 813: 112-120. doi:10.1016/S0006-8993(98)01010-5. PubMed: 9824681. 9824681

[61] Smith MA, Makino S, Kvetnansky R, Post RM (1995) Stress and glucocorticoids affect the expression of brain-derived neurotrophic factor and neurotrophin-3 mRNAs in the hippocampus. J Neurosci 15: 1768-1777. PubMed: 7891134. 7891134 10.1523/JNEUROSCI.15-03-01768.1995 PMC6578156

[62] Fuchikami M, Yamamoto S, Morinobu S, Takei S, Yamawaki S (2010) Epigenetic regulation of BDNF gene in response to stress. Psychiatry Investig 7: 251-256. doi:10.4306/pi.2010.7.4.251. PubMed: 21253408. PMC3022311 21253408

[63] Mikkelsen JD, Søderman A, Kiss A, Mirza N (2005) Effects of benzodiazepines receptor agonists on the hypothalamic-pituitary-adrenocortical axis. Eur J Pharmacol 519: 223-230. doi:10.1016/j.ejphar.2005.06.049. PubMed: 16125698. 16125698

[64] Mikkelsen JD, Bundzikova J, Larsen MH, Hansen HH, Kiss A (2008) GABA regulates the rat hypothalamic-pituitary-adrenocortical axis via different GABA-A receptor alpha-subtypes. Ann N Y Acad Sci 1148: 384-392. doi:10.1196/annals.1410.044. PubMed: 19120132. 19120132

[65] Castrén E, Rantamäki T (2010) The role of BDNF and its receptors in depression and antidepressant drug action: Reactivation of developmental plasticity. Dev Neurobiol 70: 289-297. doi:10.1002/dneu.20758. PubMed: 20186711. 20186711

[66] Sun Y, Evans J, Russell B, Kydd R, Connor B (2013) A benzodiazepine impairs the neurogenic and behavioural effects of fluoxetine in a rodent model of chronic stress. Neuropharmacology 72: 20-28. doi:10.1016/j.neuropharm.2013.04.021. PubMed: 23639432. 23639432

[67] Wu X, Castrén E (2009) Co-treatment with diazepam prevents the effects of fluoxetine on the proliferation and survival of hippocampal dentate granule cells. Biol Psychiatry 66: 5-8. doi:10.1016/j.biopsych.2009.01.023. PubMed: 19251245. 19251245

[68] Bosmann HB, Penney DP, Case KR, Averill K (1980) Diazepam receptor: specific nuclear binding of [3H]flunitrazepam. Proceedings of the National Academy of Sciences U S A 77: 1195-1198. doi:10.1073/pnas.77.2.1195. PMC348452 6102385

[69] Dalezios Y, Matsokis N (1993) Nuclear benzodiazepine binding: possible interaction with thyroid hormone receptors. Neurochem Res 18: 305-311. doi:10.1007/BF00969087. PubMed: 8479599. 8479599

[70] Saha B, Mukherjee A, Santra CR, Chattopadhyay A, Ghosh AN et al. (2009) Alprazolam intercalates into DNA. J Biomol Struct Dyn 26: 421-429. doi:10.1080/07391102.2009.10507257. PubMed: 19108581. 19108581

[71] Chung CW, Coste H, White JH, Mirguet O, Wilde J et al. (2011) Discovery and characterization of small molecule inhibitors of the BET family bromodomains. J Med Chem 54: 3827-3838. doi:10.1021/jm200108t. PubMed: 21568322. 21568322

[72] Filippakopoulos P, Qi J, Picaud S, Shen Y, Smith WB et al. (2010) Selective inhibition of BET bromodomains. Nature 468: 1067-1073. doi:10.1038/nature09504. PubMed: 20871596. 20871596 PMC3010259

[73] Kouzarides T (2007) Chromatin modifications and their function. Cell 128: 693-705. doi:10.1016/j.cell.2007.02.005. PubMed: 17320507. 17320507

[74] Zhou Z, Hong EJ, Cohen S, Zhao WN, Ho HY et al. (2006) Brain-specific phosphorylation of MeCP2 regulates activity-dependent Bdnf transcription, dendritic growth, and spine maturation. Neuron 52: 255-269. doi:10.1016/j.neuron.2006.09.037. PubMed: 17046689. 17046689 PMC3962021

[75] Baba A, Okumura S, Mizuo H, Iwata H (1983) The inhibition of diazepam and gamma-aminobutyric acid of depolarization-induced release of [^14^C]cysteine sulfinate and [^3^H]glutamate in rat hippocampal slices. J Neurochem 40: 280-284. doi:10.1111/j.1471-4159.1983.tb12683.x. PubMed: 6129289. 6129289

[76] Fedele E, Ansaldo MA, Varnier G, Raiteri M (2000) Benzodiazepine-sensitive GABAA receptors limit the activity of the NMDA/NO/Cyclic GMP pathway: a microdialysis study in the cerebellum of freely moving rats. J Neurochem 75: 782-787. PubMed: 10899955. 10899955 10.1046/j.1471-4159.2000.0750782.x

[77] Khan GM, Smolders I, Ebinger G, Michotte Y (2000) Flumazenil prevents diazepam-elicited anticonvulsant action and concomitant attenuation of glutamate overflow. Eur J Pharmacol 407: 139-144. doi:10.1016/S0014-2999(00)00720-2. PubMed: 11050301. 11050301

[78] Morgan JI, Cohen DR, Hempstead JL, Curran T (1987) Mapping patterns of c-fos expression in the central nervous system after seizure. Science 237: 192-197. doi:10.1126/science.3037702. PubMed: 3037702. 3037702

[79] Moore AN, Waxham MN, Dash PK (1996) Neuronal activity increases the phosphorylation of the transcription factor cAMP response element-binding protein (CREB) in rat hippocampus and cortex. J Biol Chem 271: 14214-14220. doi:10.1074/jbc.271.24.14214. PubMed: 8662977. 8662977

[80] Ichisaka S, Katoh-Semba R, Hata Y, Ohshima M, Kameyama K et al. (2003) Activity-dependent change in the protein level of brain-derived neurotrophic factor but no change in other neurotrophins in the visual cortex of young and adult ferrets. Neuroscience 117: 361-377. doi:10.1016/S0306-4522(02)00771-6. PubMed: 12614676. 12614676

[81] Berninger B, Marty S, Zafra F, da Penha Berzaghi M, Thoenen H et al. (1995) GABAergic stimulation switches from enhancing to repressing BDNF expression in rat hippocampal neurons during maturation in vitro. Development 121: 2327-2335. PubMed: 7671799. 7671799 10.1242/dev.121.8.2327

[82] Raivio N, Tiraboschi E, Saarikoski ST, Castrén E, Kiianmaa K (2012) Brain-derived neurotrophic factor expression after acute administration of ethanol. Eur J Pharmacol 687: 9-13. doi:10.1016/j.ejphar.2012.04.021. PubMed: 22546227. 22546227

